# Supplementary material for: Exploration of the factors related to self-efficacy among psychiatric nurses
Source: PLoS One. 2020 Apr 2;15(4):e0230740. doi: 10.1371/journal.pone.0230740 (PMC7117702; doi:10.1371/journal.pone.0230740)
Supplement: Attachment — Submitted filename: PONE-D-19-34276_reviewer TM.pdf [file pone.0230740.s002.pdf]

PLOS ONE

Exploratory study of factors influencing self-efficacy among psychiatric nurses

--Manuscript Draft--

**Manuscript Number:**	PONE-D-19-34276
**Article Type:**	Research Article
**Full Title:**	Exploratory study of factors influencing self-efficacy among psychiatric nurses
**Short Title:**	Psychiatric nurses' self-efficacy
**Corresponding Author:**	Hironori Yada<br>Yamaguchi Daigaku Igakubu Daigakuin Igakukei Kenkyuka<br>Ube, JAPAN
**Keywords:**	psychiatric nurse; self-efficacy; mental health; occupational health
**Abstract:**	This cross-sectional study aimed to clarify the distinct factors related to psych[illegible]c nurses' self-efficacy. Twenty-four items related to improved self-efficacy and 25 items related to decreased self-efficacy were examined. Participants included 132 nurses and assistant nurses, mean age (SD) was 39.73 (9.75) years, including 58 men, 73 women, and 1 unkno[illegible] Factors that improved—"Positive reactions given from patients,""Ability to be positive change in nurse-patient relationship"and "Practicability of appropriate nursing"—and decreased—"Uncertainty in psychiatric nursing" and "Nurses' role loss"—self-efficacy were extracted. Although reliability was acceptable, some factors were only weakly correlated with the Japanese version of the Generalized Self-Efficacy scale; therefore, it is possible that unique facets [illegible]elf-efficacy were measured as opposed to general self-efficacy. Thus, it is necessary to develop a scale to evaluate psychiatry nurses' self-efficacy in more detail.
**Order of Authors:**	Hironori Yada<br>Hiroshi Abe<br>Ryo Odach<br>Keiichiro Adachi
**Additional Information:**	
**Question**	**Response**
**Financial Disclosure**<br><br>Enter a financial disclosure statement that describes the sources of funding for the work included in this submission. Review the submission guidelines for detailed requirements. View published research articles from *PLOS ONE* for specific examples.<br><br>This statement is required for submission and **will appear in the published article** if the submission is accepted. Please make sure it is accurate.	This work was supported by JSPS KAKENHI(https://www.jsps.go.jp/j-grantsinaid/) [grant number 19K19498]. The supporting source had no involvements for this study design (collection, analysis, and interpretation of data), writing the report and the decision to submit the report for publication.

**Unfunded studies**<br>Enter: *The author(s) received no specific funding for this work.*<br><br>**Funded studies**<br>Enter a statement with the following details:<br>• Initials of the authors who received each award<br>• Grant numbers awarded to each author<br>• The full name of each funder<br>• URL of each funder website<br>• Did the sponsors or funders play any role in the study design, data collection and analysis, decision to publish, or preparation of the manuscript?<br>• **NO** - Include this sentence at the end of your statement: *The funders had no role in study design, data collection and analysis, decision to publish, or preparation of the manuscript.*<br>• **YES** - Specify the role(s) played.<br><br>* typeset	
**Competing Interests**<br><br>Use the instructions below to enter a competing interest statement for this submission. On behalf of all authors, disclose any competing interests that could be perceived to bias this work—acknowledging all financial support and any other relevant financial or non-financial competing interests.<br><br>This statement **will appear in the published article** if the submission is accepted. Please make sure it is accurate. View published research articles from *PLOS ONE* for specific examples.	The authors have declared that no competing interests exist.

Powered by Editorial Manager® and ProduXion Manager® from Aries Systems Corporation

**NO authors have competing interests**<br><br>Enter: *The authors have declared that no competing interests exist.*<br><br>**Authors with competing interests**<br><br>Enter competing interest details beginning with this statement:<br><br>*I have read the journal's policy and the authors of this manuscript have the following competing interests: [insert competing interests here]*<br><br>* typeset	
**Ethics Statement**<br><br>Enter an ethics statement for this submission. This statement is required if the study involved:<br><br>• Human participants<br>• Human specimens or tissue<br>• Vertebrate animals or cephalopods<br>• Vertebrate embryos or tissues<br>• Field research<br><br>Write "N/A" if the submission does not require an ethics statement.<br><br>General guidance is provided below. Consult the submission guidelines for detailed instructions. **Make sure that all information entered here is included in the Methods section of the manuscript.**	This study was approved by the Ethics Review Board of Yamaguchi University Graduate School of Medicine, School of Health Sciences (approval no. 400).<br>The participants were informed of the aim of the investigation, and written consent was obtained. The data were analyzed anonymously.

Powered by Editorial Manager® and ProduXion Manager® from Aries Systems Corporation

**Format for specific study types**<br><br>**Human Subject Research (involving human participants and/or tissue)**<br>• Give the name of the institutional review board or ethics committee that approved the study<br>• Include the approval number and/or a statement indicating approval of this research<br>• Indicate the form of consent obtained (written/oral) or the reason that consent was not obtained (e.g. the data were analyzed anonymously)<br><br>**Animal Research (involving vertebrate animals, embryos or tissues)**<br>• Provide the name of the Institutional Animal Care and Use Committee (IACUC) or other relevant ethics board that reviewed the study protocol, and indicate whether they approved this research or granted a formal waiver of ethical approval<br>• Include an approval number if one was obtained<br>• If the study involved *non-human primates*, add *additional details* about animal welfare and steps taken to ameliorate suffering<br>• If anesthesia, euthanasia, or any kind of animal sacrifice is part of the study, include briefly which substances and/or methods were applied<br><br>**Field Research**<br><br>Include the following details if this study involves the collection of plant, animal, or other materials from a natural setting:<br>• Field permit number<br>• Name of the institution or relevant body that granted permission	
**Data Availability**<br><br>Authors are required to make all data underlying the findings described fully available, without restriction, and from the time of publication. PLOS allows rare exceptions to address legal and ethical concerns. See the PLOS Data Policy and FAQ for detailed information.	No - some restrictions will apply

Powered by Editorial Manager® and ProduXion Manager® from Aries Systems Corporation

A Data Availability Statement describing where the data can be found is required at submission. Your answers to this question constitute the Data Availability Statement and **will be published in the article**, if accepted.<br><br>**Important:** Stating 'data available on request from the author' is not sufficient. If your data are only available upon request, select 'No' for the first question and explain your exceptional situation in the text box.<br><br>Do the authors confirm that all data underlying the findings described in their manuscript are fully available without restriction?	
**Describe where the data may be found in full sentences. If you are copying our sample text, replace any instances of XXX with the appropriate details.**<br><br>• If the data are **held or will be held in a public repository**, include URLs, accession numbers or DOIs. If this information will only be available after acceptance, indicate this by ticking the box below. For example: *All XXX files are available from the XXX database (accession number(s) XXX, XXX.*).<br>• If the data are all contained **within the manuscript and/or Supporting Information files**, enter the following: *All relevant data are within the manuscript and its Supporting Information files.*<br>• If neither of these applies but you are able to provide **details of access elsewhere**, with or without limitations, please do so. For example:<br><br>*Data cannot be shared publicly because of [XXX]. Data are available from the XXX Institutional Data Access / Ethics Committee (contact via XXX) for researchers who meet the criteria for access to confidential data.*<br><br>*The data underlying the results presented in the study are available from (include the name of the third party*	Data cannot be shared publicly because of ensuring data anonymity . Data may be available from the Ethics Committee (contact viaEthics Review Board of Yamaguchi University Graduate School of Medicine, School of Health Sciences) for researchers who meet the criteria for access to confidential data.

and contact information or URL).<br>• This text is appropriate if the data are owned by a third party and authors do not have permission to share the data.<br><br>* typeset	
Additional data availability information:	Tick here if the URLs/accession numbers/DOIs will be available only after acceptance of the manuscript for publication so that we can ensure their inclusion before publication.; Tick here if your circumstances are not covered by the questions above and you need the journal's help to make your data available.

Manuscript
Click here to download Manuscript yada_et_al_manuscript.docx

Exploratory study of factors influencing self-efficacy among psychiatric nurses

Hironori Yada[1], Hiroshi Abe[2], Ryo Odachi[1], and Keiichiro Adachi[3]

[1]Department of Clinical Nursing, Yamaguchi University Graduate School of Medicine, Yamaguchi, Japan

[2]Department of Clinical Psychology, Health Sciences University of Hokkaido, Hokkaido, Japan

[3]Department of Fundamental Nursing, Yamaguchi University Graduate School of Medicine, Yamaguchi, Japan

*Corresponding author:

E-mail: yadahiro@yamaguchi-u.ac.jp (HY)

Abstract

This cross-sectional study aimed to clarify the distinct factors related to psychiatric nurses' self-efficacy. Twenty-four items related to improved self-efficacy and 25 items related to decreased self-efficacy were examined. Participants included 132 nurses and assistant nurses, mean age (SD) was 39.73 (9.75) years, including 58 men, 73 women, and 1 unknown. Factors that improved—"Positive reactions given from patients,""Ability to be positive change in nurse-patient relationship"and "Practicability of appropriate nursing"—and decreased—"Uncertainty in psychiatric nursing" and "Nurses' role loss"—self-efficacy were extracted. Although reliability was acceptable, some factors were only weakly correlated with the Japanese version of the Generalized Self-Efficacy scale; therefore, it is possible that unique facets of self-efficacy were measured as opposed to general self-efficacy. Thus, it is necessary to develop a scale to evaluate psychiatry nurses' self-efficacy in more detail.

Introduction

Albert Bandura defined self-efficacy as "judgments of how well one can execute courses of action required to deal with prospective situations" (p.122)[1]. People with high self-efficacy set goals to challenge and improve their task achievement rate; however, people with low self-efficacy tend to have fluctuation in their ways of thinking, which results in dampened spirits [2]. Workers' self-efficacy is often influenced by their performance [3]. Concerning nurses, it is difficult to set clear numerical values of past achievements, because they cannot be measured by money, as with business results. In addition, psychiatric nurses specialize in caring for patients with mental disorders, and they are known to have high

recurrence rates [4]. Therefore, psychiatric nurses' achievements that affect their self-efficacy may differ from those of general workers or other nurses.

In 2016, patients' average hospital stay in general wards was 16.2 days, while in psychiatric wards it was 269.9 days in Japan [5]. Further, the average length of stay at Japanese psychiatric hospitals is significantly longer than in other Organisation for Economic Cooperation and Development countries [6]. Consequently, nurses are more likely to give up active involvement with patients who will not be leaving the hospital [7].

Furthermore, psychiatric nursing is a stressful profession [8]. More than half of the Japanese psychiatric nurses who have experienced violence have suffered lasting negative psychological effects [9]. Stress is related to nurses' self-efficacy [10], which, among psychiatric nurses, may be low. Nonetheless, previous studies have not quantitatively clarified how self-efficacy is associated with psychiatric nurses' mental health. A qualitative study clarified 18 factors influencing self-efficacy among psychiatric nurses such as "the patients presenting a positive attitude," "Building a relationship of trust with the patients," "uncertainty in caregiving," and "sense of loss regarding one's role as a nurse" [11]. Thus, a quantitative analysis of these factors is required, which may shed light on ways to improve nurses' mental health and motivation to work as well as reduce their turnover intention.

Methods

Participants and procedure

The study participants were 147 registered nurses and assistant nurses working at public and private psychiatric hospitals in a prefecture of Japan. The principal researcher requested the cooperation of nursing directors at each hospital, in writing and verbally, and anonymous self-administered questionnaires were distributed and recovered by nursing managers in September 2016. Individual participants, all recruited at the request of the

nursing directors, submitted written informed consent and could freely decline the survey, and they were nor compensated or rewarded for participating. The survey was anonymous and only the researcher could access the data. This study was approved by the Ethics Review Board of Yamaguchi University Graduate School of Medicine, School of Health Sciences (approval no. 400).

Measures

General demographics were collected, including age, years of experience as a nurse, sex, nursing education level, and job position. The scale included 24 items assessing “improved self-efficacy” and 25 items for “decreased self-efficacy” were developed based on previously determined qualitative data [11], and responses were rating using a 5-point Likert scale from 1 (*I do not think so at all*) to 5 (*I think so*).

Additionally, the Generalized Self-Efficacy Scale (GSES)[12] that was translated to Japanese (GSES-J)[13] was used to evaluate the validity of the factors related to self-efficacy. The reliability and validity of the GSES [12,14] and the GSES-J [13] were confirmed. The GSES-J comprised one factor with 23 items, which were rated on a 5-point Likert scale ranging from 1 (*I do not think so*) to 5 (*I think so*). The Cronbach's α coefficient in this study was .88, confirming its reliability as a one-factor structure.

Statistical analyses

Means, standard deviations (*SD*), and frequency (*n*) were calculated for participants’ demographic characteristics. Ceiling effect, floor effect, kurtosis, and skewness of the items were confirmed by observing the distribution on the item scores of the factors that improve and decrease self-efficacy. The factor structure was identified using factor analysis. The maximum likelihood method was used for factor extraction; the Kaiser-Guttmann criterion was also used for determining the number of factors. While conducting the exploratory factor

analysis (EFA), the Kaiser-Meyer-Olkin (KMO) measure of sampling adequacy and Bartlett's test of sphericity (χ^2) were confirmed. The required sample size was judged by KMO measure of sampling adequacy. The appropriateness of factor analysis was judged by Bartlett's test of sphericity (χ^2). Items that showed a factor loading less than 0.40 on one factor or greater than 0.40 loading on multiple factors were deleted. After the items were deleted, the EFA was repeated. The internal consistency of the factors was calculated using a Cronbach's alpha coefficient. Pearson's correlation coefficients were calculated to confirm the validity between each factor and the GSES-J. The significance level was set at $p < .05$. IBM SPSS 24.0 was used for all analyses (Windows; SPSS, Chicago, IL, USA).

Results

Demographics

The data from 132 participants who provided written informed consent were analyzed (effective response rate = 89.7[illegible]%). Participants' mean age was 39.73 years (*SD* = 9.75; range = 21.00-64.00; two missing). The mean of nursing experience was 13.05 years (*SD* = 10.57; range =0.00- 40.00; three missing). There were 58 men (43.9%), 73 women (55.3%) and 1 unknown (0.8%). There were 11 university graduates (8.3%), two nursing junior college graduates (1.5%), 112 nursing school graduates (84.8%), six with another level of education (4.5%), and one unknown (0.8%). Regarding job positions, 22 were managers (16.7%), 104 were non-managers (78.8%), and 6 were unknown (4.5%).

Factors related to self-efficacy among psychiatric nurses

The number of missing answers in each item was 0–4, which we judged as small. Thus, the mean value was substituted for the missing data in statistical analyses. Normal distribution was assumed because the skewness and kurtosis values did not exceed ±2 [15]. Three items relating to decreased self-efficacy had a ceiling effect at + 1 SD and were deleted

from the analyses. Three factors including13 items that focused on improved self-efficacy (Table 1), and two factors with 12 items that were related to decreased self-efficacy (Table 2) were ultimately extracted by factor analysis.

Table 1. Factors Related to Improved Self-efficacy Among Psychiatric Nurses (n=132)

Item no.	Item content	Factor 1	Factor 2	Factor 3
**Factor 1: Positive reactions of patients (Cronbach's α = 0.80)**				
15	Patients express their gratitude	**0.88**	-0.09	-0.02
14	By being considerate to patients, I feel that they also express their gratitude in return	**0.69**	-0.02	-0.01
12	By being considerate to patients, I can see their smiling faces	**0.58**	0.06	0.10
16	I feel that patients who are usually negative open their hearts to me	**0.50**	0.18	0.16
**Factor 2: Ability to be positive change in nurse-patient relationship (Cronbach's α: 0.84)**				
18	I can get patients to understand by explaining things	-0.09	**0.89**	-0.08
19	I can build trusting relationships with patients	0.24	**0.81**	-0.18
21	I can make patients happy	0.23	**0.58**	0.04
17	I can improve patients' opinions about rehabilitation	0.02	**0.51**	0.35
22	I feel that, among nurses of the same rank, I am relied upon	-0.23	**0.45**	0.35
**Factor 3: Practicability of appropriate nursing (Cronbach's α coefficient: 0.71)**				
2	I can anticipate symptoms and care	0.13	-0.16	**0.75**
4	I can practice nursing that I think is correct	-0.12	0.21	**0.59**
2	I can make use of training	0.04	-0.04	**0.56**

6	I feel patients' recovery goes as anticipated	0.10	-0.06	**0.53**
	Factor correlations			
	Factor 1	--		
	Factor 2	.60	--	
	Factor 3	.49	.64	--

Table 2. Factors Related to Decreased Self-efficacy Among Psychiatric Nurses (n=132)

Item no.	Item content	Factor 1	Factor 2
**Factor 1: Uncertainty in psychiatric nursing (Cronbach’s α: 0.86)**			
3	I have been refused by patients even after they have promised	**0.81**	-0.08
4	I have felt that I cannot communicate well with patients	**0.79**	0.08
7	I have felt uncertainty about my effect on patients	**0.77**	0.05
5	I have had to repeat the same explanation to patients	**0.74**	-0.07
10	Symptoms and treatment effects are obscure	**0.63**	0.01
2	I have had patients not participate in treatment when I tell them to	**0.58**	-0.02
**Factor 2: Nurses’ role loss (Cronbach’s α: 0.80)**			
21	I have felt that I am not needed as a nurse by patients	-0.12	**0.74**
22	Other staff members are more necessary to patients than I am	0.10	**0.72**
25	I have lost confidence in my ability as a nurse owing to failure	-0.07	**0.69**
20	I have felt that it is difficult to learn on my own	0.10	**0.62**
24	I have forgotten to greet patients as the days go by	0.09	**0.56**
15	I am anxious about my impaired judgment by the busyness of daily work	-0.10	**0.53**
	Factors correlations		
	Factor 1	--	
	Factor 2	0.29	--

During extraction, the KMO measure of sampling adequacy was 0.88, and Bartlett's test was χ^2 (df) = 704.10 (78) ($p < .001$). The first factor comprised items related to positive emotions felt by nurses from patients, which was named "Positive reactions of patients." The second factor comprised items related to the "Ability to be positive change in nurse-patient relationship." The third factor comprised items related to whether a nursing action or procedure was considered appropriate and feasible by nurses, which was named "Practicability of appropriate nursing."

During extraction of the factor that decreases self-efficacy, the KMO measure of sampling adequacy was 0.83 and Bartlett's test was χ^2 (df) = 596.23 (66) ($p < .001$). The first factor comprised items related to patients' responses that deviated from nursing expectations, which was named "Uncertainty in psychiatric nursing." The second factor comprised items related to nurses feeling a loss as to their role and perceived distance between nurses and patients, called "Nurses' role loss."

Reliability and validity of factors

Concerning reliability, for all factors Cronbach's α exceeded .70 (Table 3). Regarding validity, the validity between factor scores and GSES-J score were as follows: "Factors Related to Improved Self-efficacy (Total)," $r = 0.42$ ($p < .001$); "Positive reactions of patients," $r = .28$ ($p = .001$); "Positive change in nurse–patient relationship," $r = .44$ ($p < .001$); "Practicability of appropriate nursing," $r = .40$ ($p < .001$); "Factors Related to Decreased Self-efficacy (Total)," $r = -0.27$ ($p = .002$); "Uncertainty in psychiatric nursing," $r = -.05$ ($p = .55$); and "Nurses' role loss," $r = -.40$ ($p < .001$) (Table 4).

Table 3. Internal consistency of Factors Related to Self-efficacy Among Psychiatric Nurses

	Cronbach’s α coefficient
Factors Related to Improved Self-efficacy (Total)	0.86
Positive reactions of patients	0.80
Ability to be positive change in nurse-patient relationship	0.84
Practicability of appropriate nursing	0.71
Factors Related to Decreased Self-efficacy (Total)	0.80
Uncertainty in psychiatric nursing	0.86
Nurses’ role loss	0.80

Table 4. The Factors Related to Self-efficacy and the GSES-J correlations

	GSES-J
Factors Related to Improved Self-efficacy (Total)	0.42*
Positive reactions of patients	0.28*
Ability to be positive change in nurse-patient relationship	0.44*
Practicability of appropriate nursing	0.40*
Factors Related to Decreased Self-efficacy (Total)	-0.27*
Uncertainty in psychiatric nursing	-0.05
Nurses’ role loss	-.398*

$^{*}p < 0.01$

Discussion

This study aimed to clarify psychiatric nurses' mental health by exploring the factors related to their self-efficacy. Notably, the extracted factors were similar to the qualitatively extracted factors in previous studies [16].

Examination of factors that improve self-efficacy

The KMO measure of sampling adequacy exceeded 0.8 [17] and Bartlett's test was significant, indicating acceptable results. Moderate correlations existed between "Positive reactions of patients" and "Ability to be positive change in nurse-patient relationship," which were similar concepts (Table 1). Interactions in the patient-nurse relationship may lead to improved self-efficacy among psychiatric nurses because patient satisfaction is related to nurses' self-efficacy [18]. Perceived nurse caring and satisfaction with care are strongly correlated in psychiatric care [19]. However, concerns have been raised that the lack of communication between patients and medical personnel may lead to a situation in which Japanese people are distanced from mental healthcare [20]; therefore, patients' satisfaction with mental health services may be low. Improving said relationship might lead to improved self-efficacy among psychiatric nurses.

Concerning "Practicability of appropriate nursing," inadequate communication between psychiatric patients and nurses has resulted in patients expressing anger toward nurses and feeling a lack of understanding and empathy [21]. Psychiatric nurses are stressed by their own lower psychiatric nursing ability and communication with psychiatric patients. [16]. Therefore, promoting nurses' ability to practice appropriate nursing may enhance their self-efficacy.

Examination of factors that decrease delf-efficacy

The KMO measure of sampling adequacy exceeded 0.8 [17] and Bartlett's test was significant, indicating acceptable results. Concerning the first factor—"Uncertainty in psychiatric nursing"—treatment uncertainty causes discomfort [22], and nursing appears to require certainty [23]. Psychiatric nurses engage in nursing in uncertain situations where they have queries as to their nursing abilities and different attitudes towards nursing compared to non-psychiatric nurses [16]; therefore, psychiatric nurses' self-efficacy may be more influenced by nursing uncertainty as compared to general nurses.

Concerning "Nurses' role loss," as mentioned above, the lack of communication between healthcare providers and the resulting distance between patients and nurses [20] might result in decreased self-efficacy and role loss among nurses.

Examination of the factors' reliability and validity

Concerning factor reliability, Cronbach's alpha should exceed .60, and scores greater than .95 indicate redundancy [24]; therefore, the internal consistencies of all the factors were considered acceptable.

Concerning validity, the correlations between the factors and GSES-J scores ranged from weak to moderate, except for "Uncertainty in psychiatric nursing." Due to the unique factors related to self-efficacy for psychiatry nurses, "Uncertainty in psychiatric nursing" may not grasp the self-efficacy of the psychiatry nurse and may not reflect strong self-efficacy.

Study limitations

All of the 132 participants were nurses from public and private psychiatric hospitals; therefore, the possibility of selection and sampling bias cannot be excluded. This study have cross-sectional data; therefore, a longitudinal survey using a test-retest method is required.

Conclusions

In this study, three factors related to improved self-efficacy and two factors related to

decreased self-efficacy among psychiatric nurses were extracted. The validity was quantitatively confirmed in 4 out of the 5 factors. Intervention regarding the four factors will lead to the improvement of self-efficacy for psychiatric nurses; therefore, future planned interventions should examine these identified factors further to address way to improve nurses’ self-efficacy. However, some factors were weakly correlated; thus, unique facets of self-efficacy may have been measured as opposed to general self-efficacy clarifying a need to identify those unique factors. It is necessary to develop a scale that can evaluate psychiatric nurses’ self-efficacy in more detail in the future.

Acknowledgements

We thank the psychiatric nurses for their cooperation and Yamaguchi University students who helped collect questionnaires and organize data. Furthermore, we thank Editage (www.editage.jp) for English-language editing.

References

1. Bandura A. Self-efficacy mechanism in human agency. Am Psychol. 1982;37(2):122–147. https://doi.org/10.1037/0003-066X.37.2.122.
2. Bandura A. Self-efficacy: The exercise of control. New York, NY, US: W H Freeman; 1997.
3. Lunenburg FC. Self-efficacy in the workplace: implications for motivation and performance. *International Journal of Management*. 2011;14(1):1–6.
4. Koopmans PC, Bültmann U, Roelen CA, Hoedeman R, van der Klink JJ, Groothoff JW. Recurrence of sickness absence due to common mental disorders. Int Arch Occup Environ Health. 2011;84(2):193–201. https://doi.org/10.1007/s00420-010-0540-4.

5. Ministry of Health. Labor and Welfare. [Overview of medical facility surveys and hospital reports 2016 [Internet]]. Author; 2017. Japanese. Available from https://www.mhlw.go.jp/toukei/saikin/hw/iryosd/16/dl/gaikyo.pdf
6. Organisation for Economic Cooperation and Development. OECD health statistics–frequently requested data [Internet]. Author; 2014. Available from https://www.oecd.org/els/health-systems/oecd-health-statistics-2014-frequently-requested-data.htm
7. Kimura K, Matsumura H. (2012). [Nursing in psychiatric hospital inpatient wards: An analysis of nurses' conflicts in the present situation and a proposal for improvement]. Japanese Journal of Nursing Research. 2012;35(5):87–96. Japanese. https://doi.org/10.15065/jjsnr.20121011009.
8. Yoshizawa K, Sugawara N, Yasui-Furukori N, Danjo K, Furukori H, Sato Y, et al. Relationship between occupational stress and depression among psychiatric nurses in Japan. Arch Environ Occup Health. 2016;71(1):10–15. https://doi.org/10.1080/19338244.2014.927345.
9. Inoue M, Tsukano K, Muraoka M, Kaneko F, Okamura H. Psychological impact of verbal abuse and violence by patients on nurses working in psychiatric departments. Psychiatry Clin Neurosci. 2006;60(1):29–36. https://doi.org/10.1111/j.1440-1819.2006.01457.x.
10. Grau R, Salanova M, Peiro JM. Moderator effects of self-efficacy on occupational stress. Psychol Spain. 2001;6(1):63–74.
11. Yada H, Kobayashi M, Odachi R, Yamane T. Factors relating to self-efficacy among psychiatric nurses. Journal of UOEH. 2017;39(3):229–34. Japanese. https://doi.org/10.7888/juoeh.39.229

12. Sherer M, Maddux JE, Mercandante B, Prentice-Dunn S, Jacobs B, Rogers RW. The self-efficacy scale: construction and validation. Psychol Rep. 1982;51(2):663–71. https://doi.org/10.2466/pr0.1982.51.2.663.
13. Narita K, Shimonaka Y, Nakazato K, Kawaai C, Sato S, Osada Y. [A Japanese version of the generalized self-efficacy scale]. Jpn J Educ Psychol. 1995;43(3):306–314. Japanese. https://doi.org/10.5926/jjep1953.43.3_306
14. Sherer M, Adams CH. Construct validation of the self-efficacy scale. Psychol Rep. 1983;53(3):899–902. https://doi.org/10.2466/pr0.1983.53.3.899.
15. Kunnan AJ. An introduction to structural equation modelling for language assessment research. Lang Test. 1998;15(3):295–332. https://doi.org/10.1177/026553229801500302.
16. Yada H, Abe H, Funakoshi Y, Omori H, Matsuo H, Ishida Y, et al. Development of the psychiatric nurse job stressor scale (PNJSS). Psychiatry Clin Neurosci. 2011;65(6):567–75. https://doi.org/10.1111/j.1440-1819.2011.02258.x.
17. Kaiser HF. An index of factorial simplicity. Psychometrika. 1974;39(1):31–36. https://doi.org/10.1007/BF02291575.
18. De Simone S, Planta A, Cicotto G. The role of job satisfaction, work engagement, self-efficacy and agentic capacities on nurses' turnover intention and patient satisfaction. Appl Nurs Res. 2018;39:130–140. https://doi.org/10.1016/j.apnr.2017.11.004.
19. King BM, Linette D, Donohue-Smith M, Wolf ZR. Relationship between perceived nurse caring and patient satisfaction in patients in a psychiatric acute care setting. J Psychosoc Nurs Ment Health Serv. 2019;57(7):29–38. https://doi.org/10.3928/02793695-20190225-01.

20. Kanehara A, Umeda M, Kawakami N. Barriers to mental health care in Japan: results from the World Mental Health Japan Survey. Psychiatry Clin Neurosci. 2015;69(9):523–533. https://doi.org/10.1111/pcn.12267.
21. Stewart D, Burrow H, Duckworth A, Dhillon J, Fife S, Kelly S, et al. Thematic analysis of psychiatric patients' perceptions of nursing staff. Int J Ment Health Nurs. 2015;24(1):82–90. https://doi.org/10.1111/inm.12107.
22. Franks V. Evidence-based uncertainty in mental health nursing. J Psychiatr Ment Health Nurs. 2004;11(1):99–105. https://doi.org/10.1111/j.1365-2850.2004.00694.x.
23. Barker PJ. Chaos and the way of Zen: psychiatric nursing and the 'uncertainty principle.' J Psychiatr Ment Health Nurs. 1996;3(4):235–243. https://doi.org/10.1111/j.1365-2850.1996.tb00117.x.
24. Hulin C, Netemeyer R, Cudeck R. Can a reliability coefficient be too high? J Consum Psychol. 2001;10(1-2):55–69.https://doi.org/10.1207/S15327663JCP1001&2_05.

Click here to access/download
Supporting Information
yada_et_al._Authors_Contributions.docx

Click here to access/download
Supporting Information
yada_et_al._Disclosures.docx
