# Supplementary material for: Exploration of the factors related to self-efficacy among psychiatric nurses
Source: PLoS One. 2020 Apr 2;15(4):e0230740. doi: 10.1371/journal.pone.0230740 (PMC7117702; doi:10.1371/journal.pone.0230740)
Supplement: Attachment — Submitted filename: renamed_ef947.docx [file pone.0230740.s003.docx]

I would like to express my gratitude to the editor and reviewers for their insightful comments and suggestions. I have revised the manuscript point by point to reflect the suggestions of the reviewers. The manuscript has also been checked again to ensure use of native English.

|  | Review Comment | Author Response |
| --- | --- | --- |
| Reviewer #1 |  |  |
| 1 | The title need editing as does reflect the aim of the study I do suggest to read "Exploration of the factors that influence self-efficacy among psychiatric nurses" | I have revised the title as “Exploration of factors related to self-efficacy among psychiatric nurses” |
| 2 | The abstract seems incomplete.  The background and methods lacks some key information  Results are well presented through need some editing  The discussion need to discuss the key findings and avoid repletion of result presentations  The conclusion should be drawn in relationship to the aim of the study  Refences need to updated.  The details are prrovide insitu (in the manuscript). | <abstract>  I have added some information to the introductory part of the abstract. The added text is in red.  I have removed the demographics of mean age and sex and description of scale development to the main findings part.  I have revised the discussion and conclusion, as can be seen in the red font.  Updates or additions have been made to many references, except for statistics, Bandura, and literature on the scale used. |
| 3 | The abstract doesn't provide a background for readers to note the reason for the study | I have added text about the background to the study. |
| 4 | What it means by unknown. some clarification might be needed for clarity | The “unknown” referred to are the missing values. However, I have deleted this and moved it to the main findings |
| 5 | The link between the title an the findings seems sounds unclear. Is this study aiming at validation of instrument of exploration of factors that influence self-efficacy among nurses? By answering this question will lead into a focused conclusion. | The study is aimed to explore factors related to self-efficacy among nurses. The items in the questionnaire capture the self-efficacy of psychiatric nurses (Yada 2017).  Along with this, I have revised the theme to clarify the link between the theme and results. |
| 6 | Authors are required to provide the linkage between Hospital stay and self-efficacy for nurses. As it reads by now there is no appparent reason to the stated association. | I have added the necessary description linking hospital stay and self-efficacy. |
| 7 | Why the participants were recruited through the nursing directors. Can the authors descrbe the potential ethical issues related to such recruitment? | This is a misunderstanding. Nursing directors only distributed the questionnaires to each ward and collected them. Each participant was given an envelope and the questionnaires were sealed. Therefore, confidentiality was maintained.  I have revised this part to remove the misunderstanding. |
| 8 | What was used to collect the general demographic information? | The general demographic information collected included age, years of experience as a nurse, gender, nursing education level, and job position of the participants. |
| 9 | This response rate should be indicated together with the denominator. | 147 participants represent the denominator. |
| 10 | The unknown should clearly described. Multiple interpretation can result from such description eg. Didn't mention their positions? or Didn't know their position? Have unknown roles? etc. | The unknown here refers to those items left unanswered. |
| 11 | The Validity indexes are missing RMSEA, CFI etc. can be analyzed to complete the validity | The aim of this study was to explore of the factors related to self-efficacy among psychiatric nurses. Thus, I have not performed confirmatory factor analysis.  Inability to perform confirmatory factor analysis and its necessity are mentioned in the study limitations. |
| 12 | I do suggest each Table to have independent description rather than having a cluster. | As suggested, I have separated the cluster into reliability and validity. |
| 13 | How about other limitations:  1. sample size  2 Ethical issues e.g use of nurse director  Missing data etc. | I have described the sample size as mentioned earlier. The role of the nursing directors and missing data have also been explained earlier. |
| 14 | Please, update the references as some seems to be too old. | Updates or additions have been made to many references, except for statistics, Bandura, and literature on the scale used. |
| Reviewer #2 |  |  |
| 1 | It seems that the present manuscript present the results of a study related to development and psychometric assessment of psychiatric nurses self efficacy, but title and abstract does not convey this issue to readers. So it is suggested to revise title and abstract to be more informative about the topic. It is better to add some numerical findings of study in abstract too. | This cross-sectional study aimed to explore the distinct factors related to psychiatric nurses’ self-efficacy. Thus, this was revised in the research significance and in the aim of abstract. The background has been added. I believe that the results of this study are not necessarily an assessable tool, because one factor, “Uncertainty in psychiatric nursing,” was not valid.  I have only added Cronbach’s α. |
| 2 | Introduction does not explain the importance of present research sufficiently. The other point which might need authors' consideration is that the preliminary version of the developed scale was not assessed using face and content validity. | I have added text to underline the importance of the study in the introduction. Examination of content and face validity has been described in Measures on Methods. |
| 3 | In extracting the factors and identifying the number of factors, scree plot might be useful. discussion does not cover all aspects of findings, it is too brief. also in reporting the the results it is better to report the variance of self efficacy explained by this scale.  Conclusion is not provided based on present findings (line 192-195). | Screed plots have been shown as Fig1 and Fig2. Explanation has also been provided on Factors related to self-efficacy among psychiatric nurses in the Results section.  Conclusion has been provided based on present findings. |
| Reviewer #3 |  |  |
| 1 | Please explain how to calculate the sample size. | Sample size calculation has been described in the Methods. |
| 2 | Please clarify the inclusion and exclusion criteria. | Inclusion and exclusion criteria for selection of participants and the procedure adopted have been described in the Methods. |
| 3 | Is the data collection completed in 2016? if so, does it can represent the current situation? | Yes. Data collection was completed in 2016.  However, the situation surrounding psychiatric nurses has not changed significantly. For example, no changes in psychiatric care laws or significant reductions in average length of stay have been noted. Hence, I believe that the 2016 data are relevant even now. |
| 4 | What is the marital status of the nurses? I think this could influence the self-efficacy among these nurses, more details about personal information is needed. It would be better to add a demographic table. | The questionnaire did not ask for information on the marital status of the nurses. As you mention, marital status may affect self-efficacy. I have mentioned this in the section on Study limitations. Demographic tables have been added. |
| 5 | In line 69, what is the name of "The scale"? Does higher score mean better self-efficacy? It should be described in more detail. | The name of the scale used in (　) has been added.  The score means have also been described. |
| 6 | The Measuring Instrument of GSES-J also should be described in more detail. Does higher score mean better self-efficacy? | Yes. A higher score means better self-efficacy.  I have described the score means. |
| 7 | InTable1, Item 2 appears twice, but the content is different. Please confirm it. | I have confirmed that there is no duplication of items. |
| 8 | The logic and readability of the full text language need to be improved. | The manuscript has been checked by a native editor again. |
| Reviewer #4 |  |  |
| 1 | You mentioned that the purpose of the study was to identify the mental health of psychiatric nurses by exploring factors related to the self-efficacy of psychiatric nurses. However, there is a lack of logic about the relevance of self-efficiency and mental health of psychiatric nurses. The relationship between the mental health and self efficacy of psychiatric nurses should be described in the introduction part of manuscript in detail. | This is a misinterpretation. The aim was not to identify the mental health of psychiatric nurses in this study. The aim was to clarify the self-efficacy of psychiatric nurses. However, it is important to state the relationship between mental health and self-efficacy. On line 58-60, the relationship between mental health and self-efficacy has been clarified by adding a reference. |
| 2 | There was no criteria for the sample size. 132 participants is not enough to make a exploratory factor analysis for 50 questions. Among the total 50 questions, only 25 questions remained. The deleting process of items was not described. | Exploratory factor analysis was performed for Factors Related to Improved Self-efficacy and Factors Related to Decreased Self-efficacy, respectively. The sample size required for exploratory factor analysis is more than 5 times the number of items, 120 for 24 items related to improved self-efficacy and 125 for 25 related to decreased self-efficacy. This has been described in the section Sample size. Thus, I believe there are enough samples to perform exploratory factor analysis.  The process of deleting items is described in the section on Statistical analysis. |
| 3 | Moreover the correlation with the general self-efficiency scale was too low to show predictive validity. | As you say, the correlation with the general self-efficiency scale is partially low. Therefore, re-selection of items based on the results of this study is required to create a robust scale. I have mentioned this in the section, Examination of factors’ validity. |
| Reviewer #5 |  |  |
| 1 | The study examined the responses from a survey consisting of 49 items used to explain self-efficacy. However, the GSES contained 23 items and it was not clear if this was also given to each participant. The abstracts states there were a total of 49 items. | Participants answered 49 items (24 items related to improved self-efficacy and 25 items related to decreased self-efficacy). They also simultaneously answered 23 questions in GSES to measure the validity. This has been described in the Abstract. |
| 2 | The introduction needs to be revised beginning with the definition of Bandura's self-efficacy and why this is in important concept for psychiatric nurses. Nursing is a stressful profession but the authors need to be more precise about how stress affects psychiatric nurses. | As suggested, I have revised by adding two points to the Introduction. |
| 3 | Some methodological issues- The authors state that the study was anonymous but the survey were collected by the nurse manger. It is unclear whether or not this person had access to the responses or who on their staff completed the survey. This design might have placed increased pressure on participants to complete the survey and needs to be explained in the paper how the researchers protected the data. In line 57 uses the term assistant nurse. | Nursing directors only distributed the questionnaires to each ward and collected them. Each participant was given an envelope and the questionnaires were sealed. Therefore, confidentiality was ensured. I have revised these.  Nursing license is given to nurses and assistant nurses in the Japanese nursing system. The duties they perform are the same. Please note the reference.  Hironori Yada et al. Job-related stress in psychiatric assistant nurses. Nursing open 5(1) 15-20 2018.  There is no mistake in the description of an assistant nurse. |
| 4 | The role of this person needs to be defined and included in the demographic table (table 1). Table 1 should include the demographics of the sample and what type of facility the RNs worked in, e.g. academic center, community hospital, etc. This is an important table and the authors should consider what other items might need to be included. | I have added the demographic table (table 1). Since other factors, such as marital status, may affect self-efficacy, I have added “Future research should also gather information on marital status” in the Study Limitations section. |
| 5 | Results-Surveys were completed from 147 but the results states 132. The authors need to explain this discrepancy. An appendix with the survey would be helpful to the readers in analyzing the results. | The differences have been explained in detail on Line 130. |
| 6 | In the statistical section there are some results regarding the analysis. This should be in the results section. | Factor analysis has been moved to the results section. |
| 7 | A figure with the factor loadings that were significant would help clarify the results. It is easy to become confused with the factor loading tables. | Significant factor loading is shown in bold, in Table 1 and Table 2. |
| 8 | Discussion-The discussion should be revised to be stronger with the main outcome of the study -i.e. the important factors that impacted self-efficacy. This should be compared with other Japanese studies of nurses' self-efficacy and how it is different, the same in this study. | As mentioned by you, I have revised the discussion. There is only one paper in the literature that examined the self-efficacy of nurses. The results of this study were compared to that of the previous study. |
| 9 | The leading sentence in the discussion is confusing-This study aimed to clarify psychiatric nurses' mental health by exploring the factors related to their self-efficacy. Do the authors really mean this study was designed to assess the mental health of psychiatric nurses? | This cross-sectional study aimed to explore the distinct factors related to psychiatric nurses’ self-efficacy. Thus, this was revised in the research significance and in the aim of abstract and the background sections. |
| 10 | The second sentence in the discussion should be expanded Notably, the extracted factors were similar to the qualitatively extracted factors in previous studies. Explain what factors and what studies. This is probably the most important part of the paper and needs to very clear. | As you suggest, I have added the required explanation. |
| 11 | Clarification needed:  The number of missing answers in each item was 0-4, which we judged as small. (line 106).What does this mean? | 0-4 means missing values from 0 to 4. I believe a subsequent analysis would be possible by substituting the average value. I have described this in the beginning in Factors related to self-efficacy among psychiatric nurses. |
| 12 | This study have cross-sectional data; therefore, a longitudinal survey using a test-retest method is required (Line 188). The authors need to correct all grammar errors and wrong tense of verbs-e.g. line 63 not . See above line 188 "have". | I have revised from “have” to “had”. Our manuscript has been checked again for native writing. |
| 13 | Organisation for Economic Cooperation and Development countries-(line 188).Explain what is the purpose of this organization. Scientists reading this from other countries might not know. Spelling of organization-both in the text and references. | The OECD aims to contribute to economic growth, trade liberalization, and assistance to developing countries through free exchange of opinions and information between developed countries. I have supplemented the OECD description in the Introduction. |
